# Evaluation of Inflammatory Markers and Clinical Outcomes in COVID-19 Patients with Concurrent *Clostridioides difficile* Infection: A Comparative Cohort Analysis

**DOI:** 10.3390/biomedicines13010111

**Published:** 2025-01-06

**Authors:** Flavia Ignuta, Adrian Vlad, Teodor Cerbulescu, Stana Loredana, Felix Bratosin, Ovidiu Rosca, Lavinia Stelea, Daciana Nistor

**Affiliations:** 1 Doctoral School, “Victor Babes” University of Medicine and Pharmacy Timisoara, Eftimie Murgu Square 2, 300041 Timisoara, Romania; flavia.ignuta@umft.ro; 2 Department of Internal Medicine II, Division of Diabetes, Nutrition and Metabolic Diseases, “Victor Babes” University of Medicine and Pharmacy Timisoara, Eftimie Murgu Square 2, 300041 Timisoara, Romania; vlad.adrian@umft.ro; 3 Centre for Molecular Research in Nephrology and Vascular Disease, Faculty of Medicine, “Victor Babes” University of Medicine and Pharmacy Timisoara, Eftimie Murgu Square 2, 300041 Timisoara, Romania; 4 Department III—Microscopic Morphology, Discipline of Cellular and Molecular Biology, “Victor Babes” University of Medicine and Pharmacy, Eftimie Murgu Square 2, 300041 Timisoara, Romania; teodor.cerbulescu@umft.ro; 5 Department I, Discipline of Anatomy and Embryology, “Victor Babes” University of Medicine and Pharmacy, Eftimie Murgu Square 2, 300041 Timisoara, Romania; loredana.stana@umft.ro; 6 Department of Infectious Disease, “Victor Babes” University of Medicine and Pharmacy Timisoara, Eftimie Murgu Square 2, 300041 Timisoara, Romania; felix.bratosin@umft.ro (F.B.); ovidiu.rosca@umft.ro (O.R.); 7 Discipline of Obstetrics and Gynecology, “Victor Babes” University of Medicine and Pharmacy Timisoara, Eftimie Murgu Square 2, 300041 Timisoara, Romania; 8 Department of Functional Sciences, Physiology, Centre of Imuno-Physiology and Biotechnologies (CIFBIOTEH), “Victor Babes” University of Medicine and Pharmacy Timisoara, Eftimie Murgu Square 2, 300041 Timisoara, Romania; daciana_nistor@umft.ro

**Keywords:** systemic inflammation, co-infection, inflammatory markers, clinical outcomes, predictive biomarkers

## Abstract

Background and Objectives: Co-infection with *Clostridioides difficile* (*C. difficile*) in COVID-19 patients has emerged as a clinical challenge associated with increased morbidity and mortality. While both infections elicit systemic inflammation, the interplay between inflammatory markers, disease severity, and outcomes in patients with COVID-19 and concurrent *C. difficile* infection remains poorly characterized. This study aimed to evaluate the inflammatory status and clinical outcomes of patients hospitalized with COVID-19, with and without *C. difficile* co-infection, and to identify the inflammatory markers most predictive of severe disease. Methods: We conducted a retrospective cohort study of 200 hospitalized adults with confirmed COVID-19, of whom 92 had laboratory-confirmed *C. difficile* infection. Baseline demographic data, comorbidities, inflammatory markers (C-reactive protein [CRP], interleukin-6 [IL-6], ferritin, neutrophil-to-lymphocyte ratio [NLR], platelet count, albumin, and derived indices such as the CRP-to-Albumin Ratio [CAR] and Prognostic Nutritional Index [PNI]) were recorded. Clinical outcomes included ICU admission, need for mechanical ventilation, length of stay, and in-hospital mortality. Results: Patients with COVID-19 and *C. difficile* co-infection had significantly elevated inflammatory markers (CRP, IL-6, NLR) and higher CAR, alongside lower PNI, compared to those with COVID-19 alone (*p* < 0.001). Inflammatory indices correlated strongly with disease severity: elevated CAR and low PNI were associated with higher odds of ICU admission and mortality (*p* < 0.001). Multivariate analysis identified co-infection status, increased IL-6, and elevated CAR as independent predictors of severe outcomes. Conclusions: *C. difficile* co-infection in COVID-19 patients is associated with an intensified inflammatory response and worse clinical outcomes. Among the evaluated markers, CAR and PNI emerged as robust predictors of severe disease. Timely recognition of *C. difficile* co-infection and use of targeted anti-inflammatory and supportive therapies may improve patient management. Future studies should expand on these findings to optimize care and guide therapeutic strategies.

## 1. Introduction

The COVID-19 pandemic, instigated by the severe acute respiratory syndrome coronavirus 2 (SARS-CoV-2), has exerted a profound and enduring impact on global healthcare infrastructures, precipitating substantial morbidity and mortality worldwide [1,2]. As of the data up to October 2023, SARS-CoV-2 has been responsible for over 770 million confirmed cases and approximately 6.9 million deaths globally [3,4]. Although the acute phase of the pandemic has subsided, transitioning from a pandemic to an endemic phase, COVID-19 remains highly pertinent due to recurrent outbreaks and persistent community transmission, particularly in the context of emerging variants that continue to challenge public health responses [5,6].

In parallel with the sustained prevalence of COVID-19, *Clostridioides difficile* infection (CDI), a formidable nosocomial pathogen traditionally associated with antibiotic usage and resultant gut microbiota dysbiosis, has surfaced as a significant co-infection concern among COVID-19 patients [7,8,9]. Recent epidemiological studies indicate that the incidence of CDI in COVID-19 patients ranges between 5% to 15%, markedly higher than in non-COVID hospitalized cohorts [10,11]. This increased susceptibility is attributed to multiple factors, including prolonged hospitalization, increased antibiotic exposure, immunomodulatory treatments such as corticosteroids and tocilizumab, and the intrinsic immunological perturbations induced by SARS-CoV-2 infection [12,13].

The intersection of COVID-19 and CDI is underscored by their converging pathophysiological pathways, particularly concerning inflammation and immune dysregulation [14]. Severe COVID-19 is frequently characterized by a hyperinflammatory state, often termed a “cytokine storm,” which entails elevated levels of interleukin-6 (IL-6), C-reactive protein (CRP), and other pro-inflammatory cytokines [15,16]. Similarly, CDI elicits significant mucosal inflammation and systemic immune activation, mediated by toxins A and B, which exacerbate the host inflammatory response and can lead to sepsis and multi-organ dysfunction [17,18]. The concurrent presence of COVID-19 and CDI may potentiate systemic inflammation, disrupt gut–lung axis homeostasis, and complicate clinical management, thereby adversely influencing patient prognosis.

While extensive research has delineated the prognostic significance of inflammatory biomarkers in COVID-19, such as the CRP-to-Albumin Ratio (CAR), neutrophil-to-lymphocyte ratio (NLR), and Prognostic Nutritional Index (PNI), their prognostic utility in the context of dual infection with CDI remains underexplored [19,20,21]. Therefore, this study endeavors to evaluate the inflammatory profiles and clinical outcomes of COVID-19 patients with and without concurrent CDI. By systematically identifying and validating the inflammatory markers that most robustly predict severe outcomes in this dual-infection population, we aim to enhance clinical risk stratification and inform targeted therapeutic strategies. Ultimately, this research aspires to contribute to the optimization of patient management protocols, mitigating the compounded risks associated with COVID-19 and CDI co-infection, and thereby improving overall patient care in this complex and high-risk demographic.

## 2. Materials and Methods

### 2.1. Study Design and Population

This retrospective cohort study was conducted at a tertiary care academic hospital “Victor Babes” Hospital for Infectious Disease and Pulmonology from Timisoara, Romania, between January 2021 and December 2023. We reviewed the electronic medical records of adult patients (≥18 years) who were hospitalized with confirmed COVID-19 infection, diagnosed by real-time polymerase chain reaction (RT-PCR), and had available data on inflammatory markers. Among these patients, a subgroup had concurrent laboratory-confirmed CDI defined by positive stool PCR testing. A total of 200 patients were included: 92 patients with both COVID-19 and CDI (Co-infection Group) and 108 patients with COVID-19 alone (COVID-only Group). Patients with known inflammatory bowel disease, ongoing immunosuppressive therapy, other co-infections than COVID-19 and CDI, or incomplete laboratory or outcome data were excluded (n = 74). The local ethics committee approved the study protocol, and due to its retrospective nature, informed consent was waived.

### 2.2. Data Collection and Laboratory Measurements

Demographic data (age, sex), clinical characteristics (body mass index [BMI], comorbidities), and outcomes (ICU admission, mechanical ventilation, length of hospital stay, and in-hospital mortality) were extracted. Laboratory data included CRP, IL-6, complete blood count (for NLR and platelet count), ferritin, albumin, and serum creatinine. Inflammatory indices were computed as follows: CAR = CRP (mg/L)/Albumin (g/L); NLR = Neutrophils (×10⁹/L)/Lymphocytes (×10⁹/L); and PNI = (10 × Albumin [g/L]) + (0.005 × Lymphocyte count [×10⁹/L]). Samples were taken within 48 h of admission to ensure baseline values. All assays were performed in the hospital’s central laboratory using standardized, quality-controlled methods.

### 2.3. Inflammatory Indices and Clinical Definitions

For the severity assessment of COVID-19, we defined severe disease as the need for ICU admission or mechanical ventilation [22]. Mild-to-moderate disease included patients managed on standard wards with or without supplemental oxygen. CDI severity was defined according to standard guidelines, considering stool frequency and additional clinical parameters [23]. The primary outcome was the association between inflammatory markers and severe clinical outcomes (ICU admission, mechanical ventilation, mortality) in patients with and without CDI. Secondary outcomes included the length of hospital stay and correlations between inflammatory indices and severity markers.

### 2.4. Statistical Analysis

Data were analyzed using SPSS Statistics version 28.0 (IBM Corp., Armonk, NY, USA). Continuous variables were expressed as mean ± standard deviation and compared using the Student’s *t*-test or Mann-Whitney U test, as appropriate. Categorical variables were presented as counts and percentages and compared using the chi-square or Fisher’s exact test. Correlations between inflammatory markers and clinical outcomes were assessed using Pearson or Spearman correlation coefficients. Multivariate logistic regression analysis was employed to identify independent predictors of severe outcomes, adjusting for potential confounders (age, sex, BMI, comorbidities). A *p*-value <0.05 was considered statistically significant.

## 3. Results

### Patient Demographics

Table 1 compares baseline characteristics and outcomes of patients with both COVID-19 and *C. difficile* infection (COVID + CDI) versus those with COVID-19 alone (COVID-only). While the COVID + CDI group tended to be slightly older and have a higher BMI, these differences did not reach statistical significance. The prevalence of comorbidities such as hypertension, diabetes mellitus, and chronic kidney disease showed higher trends in the COVID + CDI group, though not achieving conventional significance. This may reflect the complex clinical profile of patients prone to CDI.

Notably, significant differences emerged in clinical outcomes. ICU admission rates were nearly doubled among COVID + CDI patients (39.1% vs. 22.2%, *p* = 0.01), and the need for mechanical ventilation was more than twice as high (26.1% vs. 12.0%, *p* = 0.008). These findings suggest that co-infection with *C. difficile* may exacerbate the clinical course of COVID-19, pushing patients towards more intensive care requirements. In-hospital mortality was also significantly elevated in the COVID + CDI group (23.9% vs. 11.1%, *p* = 0.01), underscoring the potential severity of this dual infectious burden.

Markers known to reflect systemic inflammation—CRP, IL-6, and NLR—were all significantly higher in the COVID + CDI group. CRP, a nonspecific but reliable marker of inflammation, was elevated by an average of nearly 20 mg/L in co-infected patients (87.4 vs. 68.9 mg/L, *p* < 0.001), while IL-6, a critical cytokine in COVID-19 pathophysiology, was also notably higher (62.1 vs. 48.7 pg/mL, *p* < 0.001). Ferritin, an acute-phase reactant often elevated in severe infections, was significantly greater in the COVID + CDI group (524 vs. 420 µg/L, *p* = 0.002), suggesting more intense inflammation or potentially impaired iron metabolism associated with severe disease states. In addition, a higher NLR (7.6 vs. 5.9, *p* < 0.001) indicates a shift in the white cell differential towards neutrophilia and/or lymphopenia, a pattern frequently correlated with poorer outcomes in critical illness. Interestingly, albumin levels were lower (30.4 vs. 33.8 g/L, *p* < 0.001) and platelets were reduced (205 vs. 228 ×10⁹/L, *p* = 0.004) in the COVID + CDI group. Patients with COVID + CDI displayed a significantly higher CAR (2.90 vs. 2.05, *p* < 0.001). By contrast, PNI—an index designed to capture both nutritional and immunologic competence—was significantly lower in the co-infected group (34.1 vs. 38.7, *p* < 0.001), as seen in Table 2.

**Figure 1:**
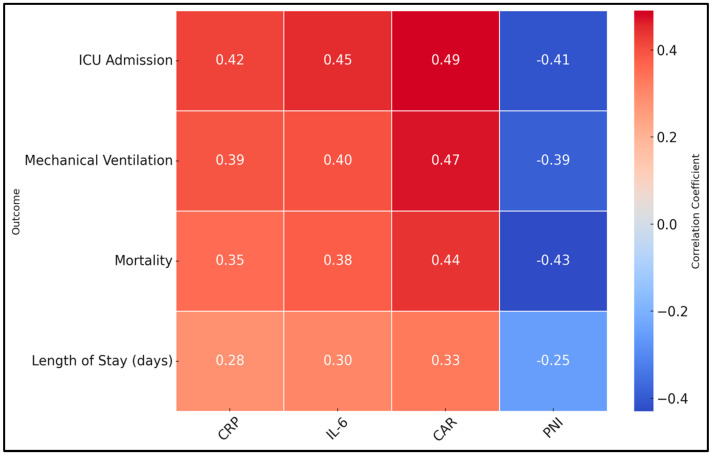
Heatmap of clinical outcomes and their correlations with inflammatory markers.

Significant positive correlations were observed between inflammatory markers (CRP, IL-6) and both ICU admission and mechanical ventilation, underscoring the role of systemic inflammation in driving severe respiratory failure and critical care needs. CAR, integrating CRP and albumin, displayed even stronger correlations with ICU admission (r = 0.49) and mechanical ventilation (r = 0.47), emphasizing its predictive strength for severe complications. Interestingly, mortality also correlated positively with CRP, IL-6, and CAR, suggesting that heightened inflammatory states may predispose patients to fatal outcomes. Conversely, PNI consistently showed negative correlations with adverse outcomes, including ICU admission (r = −0.41), mechanical ventilation (r = −0.39), mortality (r = −0.43), and length of stay (r = −0.25). This indicates that better nutritional and immunological reserves (higher PNI) may protect against progression to critical illness and death. Additionally, length of stay was positively correlated with all inflammatory markers and negatively correlated with PNI, implying that heightened inflammation and poor nutritional status may prolong hospitalization (Table 3 and Figure 1).

The comparison highlights that severe cases had significantly higher CRP (94.1 vs. 82.3 mg/L, *p* = 0.04) and IL-6 levels (67.0 vs. 58.9 pg/mL, *p* = 0.03), reinforcing the concept that escalating inflammation correlates with clinical deterioration. Additionally, CAR was meaningfully elevated in the severe group (3.10 vs. 2.77, *p* = 0.04), whereas PNI was lower (32.9 vs. 35.0, *p* = 0.03), indicating worse nutritional/immune status. The presence of more pronounced inflammation and suboptimal nutritional reserves appears to predispose to severe clinical courses. The significantly increased mortality rate (38.9% vs. 14.3%, *p* = 0.007) in the severe cohort underscores the high-stakes nature of inflammation and co-infection. Furthermore, the severe group experienced prolonged hospitalization (18.5 vs. 13.9 days, *p* = 0.001), suggesting that intense inflammation and organ support requirements extend the recovery period, as presented in Table 4.

**Figure 2:**
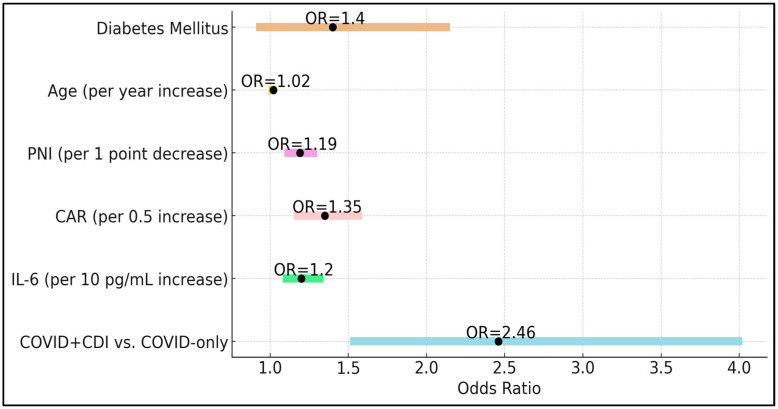
Forest plot of predictors of severe outcomes.

The presence of CDI emerged as a strong independent risk factor (OR 2.46, 95% CI 1.51–4.02, *p* < 0.001), emphasizing that beyond pre-existing conditions, co-infection with CDI significantly worsens the clinical trajectory of COVID-19. Elevations in IL-6 increased the odds of severe outcomes by 20% for each 10 pg/mL increment (OR 1.20, *p* = 0.001), underscoring the pathogenic role of uncontrolled cytokine release. Similarly, each 0.5-unit increase in CAR raised the odds of severe outcomes by 35% (OR 1.35, *p* < 0.001), and each unit decrease in PNI increased the odds by 19% (OR 1.19, *p* < 0.001), highlighting the intertwined importance of inflammatory burden and nutritional status. Interestingly, age and diabetes mellitus, while clinically relevant comorbid factors, did not reach statistical significance as independent predictors in this model (*p* = 0.20 and *p* = 0.12, respectively). This finding suggests that, within a population already hospitalized for COVID-19, the dynamic interplay of acute inflammatory markers and the presence of CDI may overshadow these baseline factors (Table 5 and Figure 2).

## 4. Discussion

### 4.1. Analysis of Findings

The current study highlights the detrimental impact of CDI on the inflammatory milieu and clinical outcomes of hospitalized COVID-19 patients. We found that those with CDI had consistently higher inflammatory markers—CRP, IL-6, NLR—and elevated CAR, coupled with reduced PNI. These aberrations correlated strongly with more severe disease, higher ICU admission rates, mechanical ventilation requirements, and mortality. By demonstrating that CDI further intensifies inflammation in COVID-19, our results advance our understanding of how complex microbiological interactions shape clinical trajectories.

These findings align with previous studies documenting the prognostic significance of CAR and PNI in various critical illnesses, including COVID-19. Our data underscore that the nutritional and immunological compromise reflected by a low PNI may exacerbate vulnerability to severe disease. Similarly, a high CAR, indicating pronounced inflammation relative to serum albumin levels, portends a more aggressive clinical course. The interplay of gut and systemic inflammation in CDI likely amplifies host vulnerability, leading to more severe pulmonary and systemic manifestations of COVID-19.

By identifying independent predictors of severe outcomes, including CDI status, IL-6, CAR, and PNI, this study suggests potential avenues for therapeutic intervention. Early identification and treatment of CDI, alongside aggressive modulation of inflammation and nutritional support, may limit disease progression. Interdisciplinary care teams could leverage these biomarkers to triage patients, prioritize resources, and tailor treatments—ranging from antimicrobial stewardship and probiotic supplementation to targeted immunomodulators. Future prospective studies and clinical trials should further clarify how these interventions might improve outcomes and refine standard care protocols.

Similarly, in a retrospective analysis conducted at Mureș County Clinical Hospital in Romania, Stoian et al. evaluated the impact of COVID-19 and CDI among intensive care unit patients [24]. The study, segmented into two groups of patients—those with both SARS-CoV-2 and CDI and those with CDI alone—uncovered no significant correlations between the presence of comorbidities and the utilization of mechanical ventilation methods. However, notable statistical associations were found, including a significant correlation between renal and hepatic comorbidities, an increased mortality rate in the presence of co-infection, and a higher incidence of CDI in patients treated with fluoroquinolone and those with diabetes. In a similar manner, a study by Zouridis et al. during the same period in New York’s Capital Area highlighted an increase in CDI rates, with an incidence of 8.06 per 10,000 patient days during the pandemic, compared to 5.67 per 10,000 patient days before the pandemic [25].

Another similar study explored the consequences of CDI among patients diagnosed with COVID-19 [26]. They observed that patients co-infected with CDI had a higher mortality risk compared to those without such co-infection, with a hazard ratio of 2.6 (*p* = 0.021), which escalated dramatically to 6.5 after 20 days of disease progression (*p* < 0.001). The study also noted that CDI-associated diarrhea was more severe and prolonged than diarrhea associated with other causes in COVID-19 patients. Similarly, Awan et al.’s investigation, which utilized the 2020 National Inpatient Sample (NIS) database, reinforced these findings at a larger scale across the United States [27]. Among over 1.6 million patients evaluated, those with concurrent *C. difficile* and COVID-19 infections exhibited significantly poorer outcomes, including higher in-hospital mortality rates (23% vs. 13.4%), more frequent in-hospital complications like ileus and septic shock, and longer hospital stays. Notably, the financial burden was also substantially greater for those with co-infection.

In a study by Merchante et al., the impact of the COVID-19 pandemic on the incidence of healthcare-associated CDI was investigated in two Spanish hospitals, revealing that the incidence rate during the pandemic did not significantly increase compared to the pre-pandemic period [28]. Specifically, at Hospital Universitario de Valme, the incidence rate decreased from 4.1 to 2.6 per 10,000 occupied bed-days during the COVID-19 period, although this change was not statistically significant (*p* = 0.1). By contrast, Hospital General Universitario de Alicante showed a consistent incidence rate before and during the pandemic (3.7 vs. 3.9 per 10,000 occupied bed-days, *p* = 0.8), despite a noted increase in antibiotic consumption. In a similar manner, a study by Manea et al. in a Romanian tertiary care hospital found no significant change in CDI incidence during the pandemic compared to the pre-COVID period, with rates of 5.6 per 1000 adult discharges during the pandemic and 6.1 per 1000 pre-pandemic (*p* = 0.6) [29].

Other research from the Balkan region, in the vicinity of Romania, revealed a significant escalation in the incidence of CDI during the COVID-19 pandemic, with a steep rise from 2.65 infections per 10,000 bed-days at the onset to 13.93 by March 2022 (*p* < 0.001) [30]. This surge reflected a more pronounced increase during the pandemic compared to the pre-pandemic period, where incidence rates only rose from 0.00 to 3.36 per 10,000 bed-days. In a similar manner, a study by Maldonado-Barrueco et al. in a Madrid hospital also documented a notable rise in CDI during the pandemic [31]. The relative incidence per 1000 admissions increased across the pandemic years, with the incidence density per 10,000 patient-days also rising, highlighting a sustained and intensified prevalence of CDI from the first to the second year of the pandemic. Both studies underscore a concerning trend: despite heightened awareness and potentially rigorous infection control practices during the pandemic, the incidence of CDI has increased, suggesting that additional factors such as disruptions in normal healthcare operations or changes in antibiotic prescribing patterns may have contributed significantly to this rise.

### 4.2. Study Limitations

This study has several limitations. First, its retrospective design may predispose to selection bias and limit the establishment of causal relationships. We relied on electronic medical records from a single center, which may reduce generalizability to other populations or healthcare settings. Additionally, we focused on baseline inflammatory markers obtained upon admission; longitudinal measurements could offer more dynamic insights into how inflammation evolves and responds to treatment over time. We did not standardize therapies for COVID-19 or CDI, and variations in antibiotic administration, immunomodulators, or supportive measures may have influenced outcomes. Furthermore, this study excluded immunosuppressed patients and those with incomplete data, potentially overlooking other subpopulations at risk; therefore, it is difficult to assemble a proper control group. While we identified strong associations between inflammatory markers and outcomes, these biomarkers must be validated in larger, multicenter studies before being integrated into routine clinical practice. Despite these constraints, our findings provide valuable insights to guide future research and clinical decision-making.

## 5. Conclusions

CDI exerts a substantial negative impact on the inflammatory profile and clinical outcomes of hospitalized patients with COVID-19. The presence of CDI is associated with heightened systemic inflammation, demonstrated by elevated CRP, IL-6, and NLR, and an unfavorable balance between inflammatory drive and nutritional state, as evidenced by higher CAR and lower PNI. These alterations correlate strongly with increased severity of illness, as reflected in higher ICU admission rates, more frequent mechanical ventilation, and greater in-hospital mortality. Our findings suggest that clinicians should maintain a high index of suspicion for CDI in COVID-19 patients, particularly those presenting with pronounced inflammation and nutritional compromise.

## Figures and Tables

**Table 1:** Baseline demographic and clinical characteristics.

Variable	COVID + CDI (n = 92)	COVID-Only (n = 108)	*p*-Value
Age (years)	67.3 ± 13.1	64.9 ± 11.9	0.18
Male Gender (%)	58.7%	54.6%	0.53
BMI (kg/m^2^)	28.4 ± 4.9	27.2 ± 4.5	0.07
Hypertension (%)	76.1%	64.8%	0.09
Diabetes Mellitus (%)	51.1%	39.8%	0.1
Chronic Kidney Disease (%)	29.3%	18.5%	0.08
ICU Admission (%)	39.1%	22.2%	0.01
Mechanical Ventilation (%)	26.1%	12.0%	0.008
In-hospital Mortality (%)	23.9%	11.1%	0.01
Vaccine Status (fully vaccinated)	43, (46.74%)	68, (62.96%)	0.03
Use of Antivirals	68, (73.91%)	77, (71.30%)	0.69
Use of Systemic Anti-inflammation Agents	36, (39.13%)	33, (30.56%)	0.16

**Table 2:** Inflammatory markers on admission.

Marker	COVID + CDI (n = 92)	COVID-Only (n = 108)	*p*-Value
CRP (mg/L)	87.4 ± 27.8	68.9 ± 23.1	<0.001
IL-6 (pg/mL)	62.1 ± 19.4	48.7 ± 15.6	<0.001
Ferritin (µg/L)	524 ± 145	420 ± 132	0.002
NLR	7.6 ± 2.5	5.9 ± 2.0	<0.001
Albumin (g/L)	30.4 ± 5.2	33.8 ± 5.7	<0.001
Platelet (×10⁹/L)	205 ± 58	228 ± 61	0.004
CAR	2.90 ± 0.83	2.05 ± 0.69	<0.001
PNI	34.1 ± 4.8	38.7 ± 5.3	<0.001

CRP—C-Reactive Protein; IL-6—Interleukin-6; NLR—Neutrophil-to-Lymphocyte Ratio; CAR = CRP (mg/L)/Albumin (g/L); PNI = (10 × Albumin [g/L]) + (0.005 × Lymphocyte count [×10⁹/L]).

**Table 3:** Clinical outcomes and their correlations with inflammatory markers.

Outcome	r (CRP)	*p*-Value	r (IL-6)	*p*-Value	r (CAR)	*p*-Value	r (PNI)	*p*-Value
ICU Admission	0.42	<0.001	0.45	<0.001	0.49	<0.001	−0.41	<0.001
Mechanical Ventilation	0.39	<0.001	0.4	<0.001	0.47	<0.001	−0.39	<0.001
Mortality	0.35	<0.001	0.38	<0.001	0.44	<0.001	−0.43	<0.001
Length of Stay (days)	0.28	0.003	0.3	0.002	0.33	0.001	−0.25	0.008

**Table 4:** Subgroup analysis: severe vs. non-severe COVID-19 in patients with *C. difficile*.

Variable	Severe (n = 36) *	Non-Severe (n = 56)	*p*-Value
CRP (mg/L)	94.1 ± 28.9	82.3 ± 25.7	0.04
IL-6 (pg/mL)	67.0 ± 20.5	58.9 ± 18.4	0.03
CAR	3.10 ± 0.81	2.77 ± 0.84	0.04
PNI	32.9 ± 4.5	35.0 ± 4.8	0.03
Mortality (%)	38.90%	14.30%	0.007
Length of Stay (days)	18.5 ± 6.4	13.9 ± 5.2	0.001

ICU—Intensive Care Unit; CAR—C-Reactive Protein-to-Albumin Ratio; PNI—Prognostic Nutritional Index; *—Severe disease defined as requiring ICU or mechanical ventilation.

**Table 5:** Multivariate logistic regression for predictors of severe * outcomes.

Variables	Odds Ratio	95% CI	*p*-Value
COVID + CDI vs. COVID-only	2.46	1.51–4.02	<0.001
IL-6 (per 10 pg/mL increase)	1.2	1.08–1.34	0.001
CAR (per 0.5 increase)	1.35	1.15–1.59	<0.001
PNI (per 1-point decrease)	1.19	1.09–1.30	<0.001
Age (per year increase)	1.02	0.99–1.04	0.2
Diabetes Mellitus	1.4	0.91–2.15	0.12

*—Severe disease defined as requiring ICU or mechanical ventilation.

## Data Availability

The data presented in this study are available on request from the corresponding author.
